# Effect of Process Duration on Electrochemical Performance in Composite Cathodes for All-Solid-State Li-Ion Batteries Processed via Warm Isostatic Pressing

**DOI:** 10.1021/acsomega.5c10291

**Published:** 2025-12-24

**Authors:** Kazushi Hayashi, Takuya Mori, Chad Beamer, Hiroyuki Ito

**Affiliations:** 1 Applied Physics Research Laboratory, Kobe Steel, Ltd., 1-5-5, Takatsukadai, Nishi-ku, Kobe, Hyogo 651-2271, Japan; 2 Kobelco Research Institute, Inc., 1-5-5 Takatsukadai, Nishi-ku, Kobe, Hyogo 651-2271, Japan; 3 Quintus Technologies, LLC, 8270 Green Meadows Drive North, Lewis Center, Ohio 43035, United States; 4 New Business Development Division, Machinery Business, Kobe Steel Ltd., 2-3-1 Shinhama Arai-cho, Takasago, Hyogo 676-8670, Japan

## Abstract

Isostatic pressing is frequently used to densify various components of all-solid-state batteries (ASSBs). Among these, warm isostatic pressing (WIP) has proven to be a useful technique for ASSB production. We conducted a parametric study of the performance of composite cathodes treated with WIP to investigate the effect of the process duration on the performance of ASSBs. Electrochemical impedance spectroscopy (EIS) and charge–discharge performances of the test cells were examined to determine the interfacial properties of the composite cathodes. X-ray computed tomography (CT) using synchrotron radiation was performed before and after WIP to correlate the interfacial properties with the microstructures of the composite cathodes. Our findings showed that the test cells had specific capacities of around 125 mAh g^–1^ when the composite cathodes were treated with WIP for 60–1800 s. However, for shorter WIP treatments (1 s), it remained around 80 mAh g^–1^, which is two-thirds of those obtained from longer WIP. According to the X-ray CT analysis, the cathodes treated with one s WIP contained a large number of small voids in the solid electrolytes (SE). Such voids were eliminated during longer WIP treatments. The change in the void ratio correlated well with the EIS results, suggesting that the resistance related to charge transfer was the dominant factor determining the performance of the ASSBs. The lower-temperature WIP resulted in insufficient densification. Additional degradation modes were enhanced in the low-temperature regime, probably due to the formation of gaps and cracks. In conclusion, process duration is a crucial factor in determining the performance of composite cathodes and hence should be carefully controlled to obtain suitable interfacial properties between the active materials and SEs. The presented results give insights into the comprehension of the interface issues in the ASSBs from both scientific and industrial aspects. They contribute to further improvement of the electrochemical performance of ASSBs.

## Graphical abstract

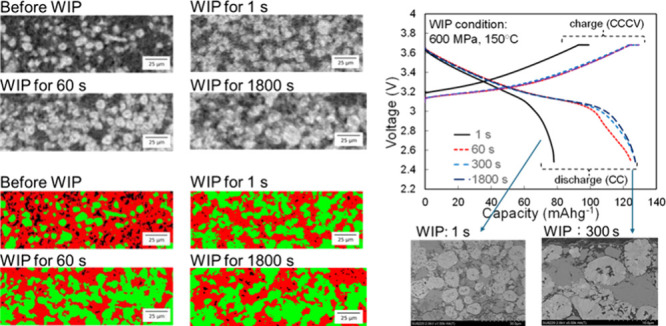

## Introduction

All-solid-state batteries (ASSBs) have attracted significant attention as high-performance Li-ion batteries (LIBs) for next-generation electric vehicles (EVs)^1–3^ because they are expected to provide higher power, a longer driving range, and higher levels of safety than conventional liquid-type LIBs.^4–11^ Efficient lithium-ion transport at the interface between the active material (AM) and solid electrolyte (SE) is essential for enhancing the performance of LIBs. However, in ASSBs, the solid nature of the electrolyte poses a challenge in establishing effective interfacial contact between the AM and SE within the composite electrodes. Hence, controlling the interfacial properties between the AM and SEs is crucial.^12–18^ In addition, the microstructure of composite cathodes severely affects the ionic conductivity of Li.^19–22^

Early work by Tatsumisago et al. showed that the Li-ion conductivity of 75Li_2_S·25P_2_S_5_ increased identically to bulk conductivity when compressed at pressures over 350 MPa.^23^ Accordingly, the relative density of 75Li_2_S·25P_2_S_5_ exceeded 90%. Further, small voids and cracks vanished from the surface of the pellets with hot pressing at 200 °C. This finding indicates that optimization of the densification process is mandatory not only for forming conformal contact at the interface but also for obtaining a higher specific power density that is sufficient for the practical use of EVs.

Isostatic pressing (IP) is recognized as a useful densification technique for ASSB production.^21,24–27^ Among these, warm isostatic pressing (WIP) is a key technology facilitating ASSB fabrication.^21,26,27^ Lee et al. reported ASSBs with high energy densities of over 900 Wh L^–1^ by using WIP.^26^ They pointed out that the WIP improved the contact properties between the electrode and electrolyte. Hayashi et al. demonstrated the application of WIP for the densification of composite cathodes and observed a drastic improvement in their electrochemical performance.^27^ They also suggested that the densification process consisted of a two-step process. The first was the rapid densification of the cathodes, which was suspended owing to physical contact with hard AM particles. Gradual densification was followed by a reduction in the number of voids within the SEs. At present, however, a pathway for obtaining composite cathodes with optimized structures remains an open question; hence, there is still room for improvement in electrochemical performance.

Electrochemical impedance spectroscopy (EIS) is a common technique widely used for characterizing the electrochemical performance of conventional LIBs and has recently been applied to ASSBs.^19,21,28–33^ By analyzing the impedance data obtained from a wide frequency range, the origins of the resistance that limit the performance of ASSBs can be determined separately. This provides clear insights for the further development of ASSBs. In this study, we conducted a parametric investigation of the densification of composite cathodes treated with WIP. Focusing on the effect of the process duration on the performance of the ASSBs, EIS and charge–discharge performances of the composite cathodes were examined to determine the limiting factors for the electrochemical performance of the ASSBs. The structure of the composite cathodes before and after WIP was examined by X-ray computed tomography (CT) using synchrotron radiation at SPring-8 to correlate the structure of the cathodes with their electrochemical performance.

## Experimental Section

### Preparation of Composite Cathodes

Commecially available Li­(Ni_1/3_Mn_1/3_Co_1/3_)­O_2_ particles were used as the AM. The surface of the AM was coated with LiNbO_3_ prepared by spray-coating with an ethanol solution containing lithium ethoxide and niobium ethoxide using a tumbling fluidized-bed granulating coater (MP-01, Powrex). The SE of the composite cathodes was argyrodite-type Li_6_PS_5_Cl (D50 ≈ 1 μm, Ampcera). They were mixed with acetylene black (AB) as a conductivity agent, with a cathode composition ratio of 74:23:3 wt %. Then, binder and solvent were added to form a slurry. Subsequently, the composite slurry was coated and dried on an Al foil that acted as a current collector. Finally, the composite cathodes were placed in laminate bags and vacuum-sealed. Only the materials with the same production batch were used to avoid uncertainties in the obtained results due to unexpected property changes with the progress of the developments by producers. The nominal thickness of the as-fabricated composite cathode was approximately 170 μm. A typical cross-sectional secondary electron microscopy (SEM) image of the as-fabricated composite cathode is shown in Figure 1a.

**Figure 1:**
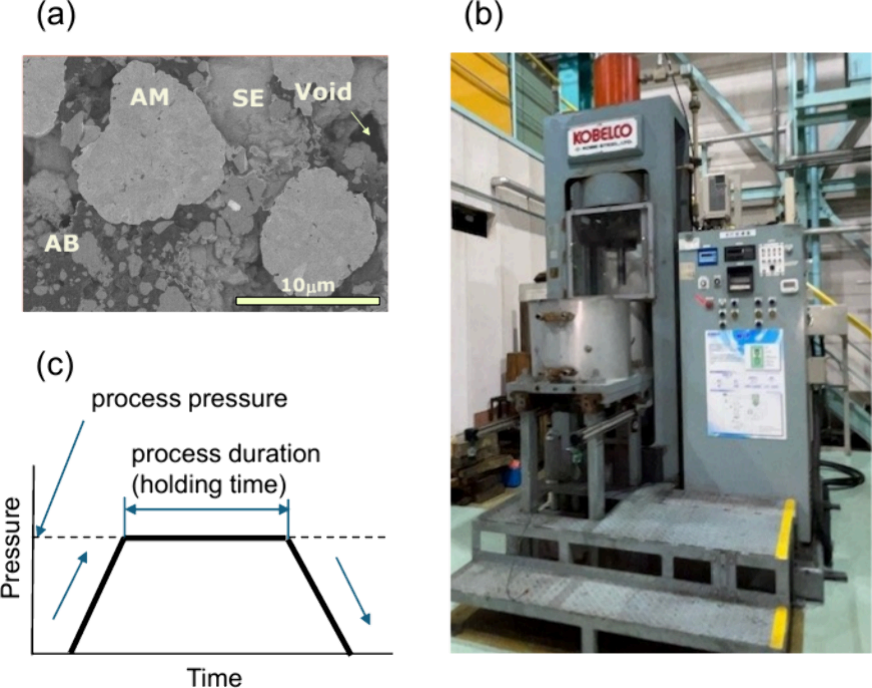
(a) Typical cross-sectional secondary electron microscopy image for as-fabricated composite cathodes; (b) WIP system used in this study; and (c) pressurizing sequence.

WIP was conducted using a piston-type vertical cold IP equipment capable of sample heating up to 190 °C during treatment [Kobe Steel, Ltd.; shown in Figure 1b]. The vessel was 80 mm in diameter and 180 mm long. The maximum processing pressure was 980 MPa. In the present study, the process pressure was maintained at 600 MPa, whereas the process duration was varied from 1 to 1800 s. The duration range was chosen so that the structural changes in the composite cathodes could be examined from the initial contact to the point at which the structure of the composites was finalized. The processing conditions for each cathode are listed in Table 1. The thickness of the composite cathodes was reduced to around 100 μm after WIP. The pressurizing sequence is schematically shown in Figure 1c. The pressure medium inside the vessel was preheated. After the medium reached the required temperature, the samples were loaded into the vessel at atmospheric pressure and pressurized by using the pressure medium. The pressure was maintained at the process pressure for a certain amount of time and then released. Hence, the process duration (sometimes termed the holding time) in this experimental sequence indicates the time elapsed from the moment the pressure in the vessel reached the maximum pressure in each experiment.

**Table 1:** Sample Description of Composite Cathodes and Test Cells Used in This Study, along with Pressing Conditions for Each Cathode

		cathode					
sample	type	WIP	pressure (MPa)	temperature (°C)	duraition (s)	electrolyte	anode
CC0	composite cathode						
CCW1	composite cathode	√	600	150	1		
CCW60	composite cathode	√	600	150	60		
CCW300	composite cathode	√	600	150	300		
CCW1800	composite cathode	√	600	150	1800		
TCW1	test cell	√	600	150	1	pellet	In–Li alloy
TCW60	test cell	√	600	150^a^	60	pellet	In–Li alloy
TCW300	test cell	√	600	150^a^	300	pellet	In–Li alloy
TCW1800	test cell	√	600	150^a^	1800	pellet	In–Li alloy

a Test cells having composite cathodes treated with WIP at 120 °C were also prepared for discussion.

### Electrochemical Performance of the Composite Cathodes

The charge–discharge performance was examined by using the test cell shown in Figure 2. To evaluate the performance of the composite cathodes, test cells were stacked with bulky pellet-type SEs, and In–Li alloys were attached to the other side of the SEs. The working electrodes were composite electrodes with and without WIP treatment. The bulky SEs were composed of Li_6_PS_5_Cl formed by uniaxial pressing at 600 MPa, and In–Li alloys were used as the counter electrodes. The effective cell area was approximately 1 cm^2^, and the thickness of the SE pellet was about 600 μm. The test cells used in this study are given in Table 1. During measurements, the test cells were confined to a pressure of 150 MPa to maintain the physical contact between the components.

**Figure 2:**
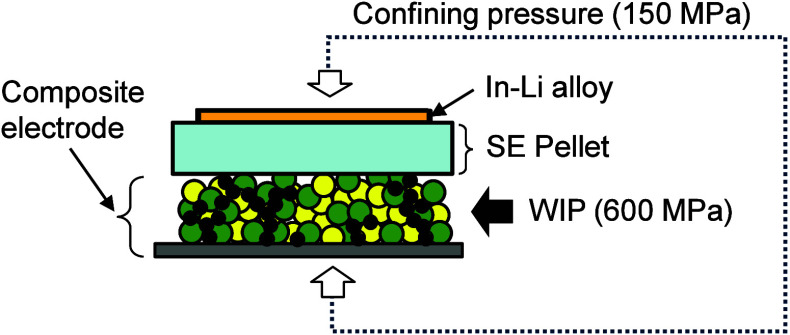
Test cells used in this study. The effective cell area is approximately 1 cm^2^, and the thickness of the pellet SE is ca. 600 μm. The test cells were pressurized to a confining pressure of 150 MPa during the evaluation.

The charge–discharge performance was evaluated at a current density of 15 mA/g (0.1C). Constant current (CC) mode was utilized for discharging. The cutoff voltage for discharge was set to 2.48 V. For charging, both CC and constant voltage (CV) modes were used. During the CV mode, the charging voltage was maintained at 3.68 V for 1 h. EIS was performed during charging in the range of 3.3–3.5 V, before charging, and after charging states for comparison. The amplitude of the small-signal input was 10 mV, and the frequency for the measurement ranged from 1 × 10^–2^ to 7 × 10^6^ Hz. Once the applied voltage reached each measurement value (from 3.3 to 3.5 V with 0.1 V increments per step), it was kept constant for 1 h to form a uniform electric field in the cathodes. Then, measurements were carried out.

### Structural Evaluation Using X-ray-Computed Tomography with Synchrotron Radiation

The structures of the composite cathodes before and after WIP were examined by X-ray CT using synchrotron radiation at SPring-8 (SUNBEAM: BL16B2). The incident X-ray beam was monochromatized and irradiated at 30 keV. The samples for the CT measurements were encapsulated in a glass capillary and purged in an Ar atmosphere. The capillary diameter was 0.8 mm. The sample rotation angle during the measurement was 180°, and the exposure time was set to 4 s. CT data were obtained with an Xsight Micron LC instrument (Rigaku Innovative Technologies Europe) every 0.2°. The effective pixel size of the experimental setup was 0.65 μm.

Based on the CT values separately determined for various elements, including AM, SE, and voids/carbon, each voxel was classified into three categories to analyze the structure of the composite cathodes. Voids and carbon-related materials were not distinguished because of their high transparency to X-rays. The void ratio, *V*, was determined using the number of voxels for carbon/voids, *N*_void_, and the number of voxels for all elements, *N*_all_, as follows:

$$V=\frac{N_{\mathrm{void}}}{N_{\mathrm{all}}}$$ 1

Some samples were characterized using cross-sectional SEM. Cross sections were prepared by cryofocused ion beam milling to avoid damage to the samples during preparation.

## Results and Discussion

Figure 3 shows the charge–discharge performance of the test cells as a function of the process duration. All of the composite cathodes were isostatically pressurized at 600 MPa and 150 °C, and the specific capacity of the test cells increased with process duration. The specific capacities of TCW60, TCW300, and TCW1800 were approximately 125, 127, and 127 mAh g^–1^, respectively. In contrast, the specific capacity of TCW1 remained at approximately 80 mAh g^–1^, while the discharging voltage of TCW1 decreased significantly with the capacity. In contrast, the discharging curves of TCW60, TCW300, and TCW1800 were identical in the low- and medium-capacity regimes, but the features of voltage decay observed over 100 mAh g^–1^ varied, and an apparent dip was observed, especially for TCW60.

**Figure 3:**
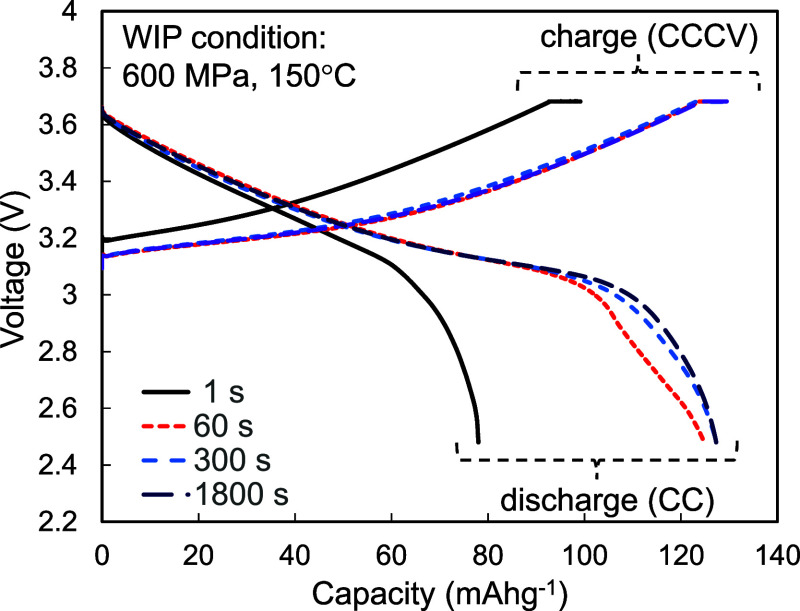
Charge–discharge performance of the test cells as a function of the process duration. All the composite electrodes were pressurized at 600 MPa and 150 °C via WIP.

Figure 4 shows the reconstructed CT images [Figure 4a–d] and corresponding categorized composition images [Figure 4e–h] of the composite cathodes before and after the WIP. Figure 4a,e shows the reconstructed CT images and corresponding categorized composition images, respectively, for the composite cathodes before WIP (CC0). A relatively large spot-like black area, which corresponds to voids and carbon-related materials, was clearly observed in the SE region (red area). The small portion of the green area (corresponding to AM) suggests that the structure of CC0 is coarse. After WIP treatments at 600 MPa and 150 °C, the spot-like black area almost diminished, as evident in Figure 4f–h for CCW60, CCW300, and CCW1800, respectively. The increase in the AM area (corresponding to the area shown in green) indicates that the composite cathodes were densified by WIP treatment. However, the microstructure of the composite cathodes varied, depending on the process duration. The void ratio *V*, defined by eq 1, was calculated, and the results are plotted in Figure 5. The void ratio for CCW1 was approximately 0.03, which is almost twice that for the longer cases (CCW60, CCW300, and CCW1800), indicating that many small voids remained in the SE region of CCW1.

**Figure 4:**
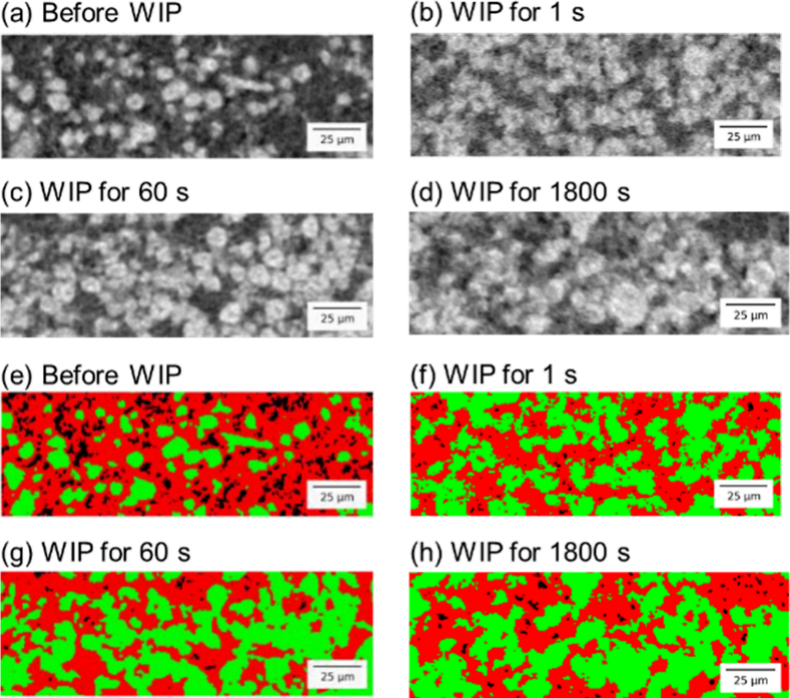
Reconstructed CT images (top) and corresponding categorized composition images (bottom) of the composite cathodes: (a), (e) CC0, (b), (f) CCW60, (c), (g) CCW300, and (d), (h) CCW1800.

**Figure 5:**
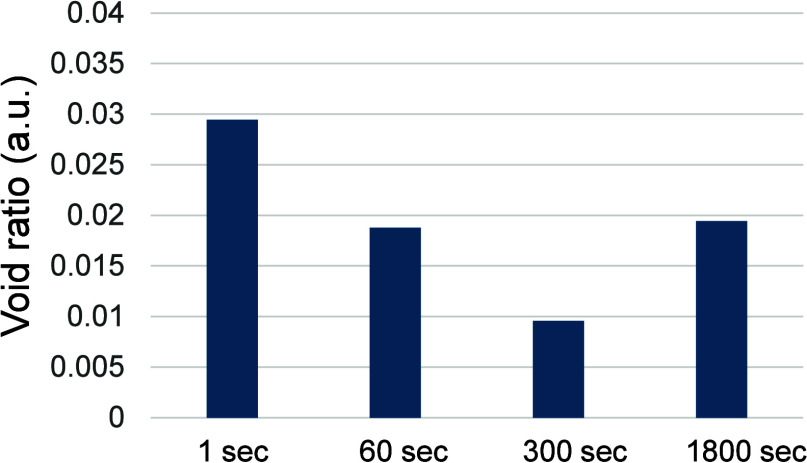
Void ratios were calculated for the CC0, CCW60, CCW300, and CCW1800 cathodes.

The EIS results for the corresponding test cells are listed in Figure 6. In the Nyquist plots obtained at a voltage of 3.3 V, the real impedance against the imaginary impedance contained two major symmetrical arcs with a large offset from the origin in the coordinate system; furthermore, the plots monotonously increased with almost the same slope. The frequency at the apex of the semicircle indicates the origin of resistance. The origins of the cathode and anode contributions to the impedance spectra in the ASSBs were extensively studied and reported as the charge transfer between the AM and SE,^28,30,33^ the In–Li anode related,^28,33^ the degradation layer formed during the operation cycles,^28^ and Li-ion intercalation/deintercalation observed in the low-temperature regime.^30^ The first arc was observed in a frequency range between 1 MHz and 1 kHz. The observation supported that the origin of the resistance was related to charge transfer. The resistance related to In–Li anodes and to the degraded layers could be excluded because the reported frequency ranges were lower and higher than the case for the charge transfer, respectively. The second arc was observed in a lower frequency range, roughly between 1 kHz and 1 Hz, with a considerable charging voltage dependence (see Figure 8). This fact suggested that the R3 was mainly dominated by the phenomena at the anode/SE interfaces,^31^ although the contribution of Li-ion intercalation/deintercalation^30^ could not be excluded. The R3 mainly originated from the anode side; however, the variation of the R3 should be attributed to the structural changes in the cathodes, since the contribution of the anode-related resistance was common to all the test devices having identical structures except for their densification conditions.

**Figure 6:**
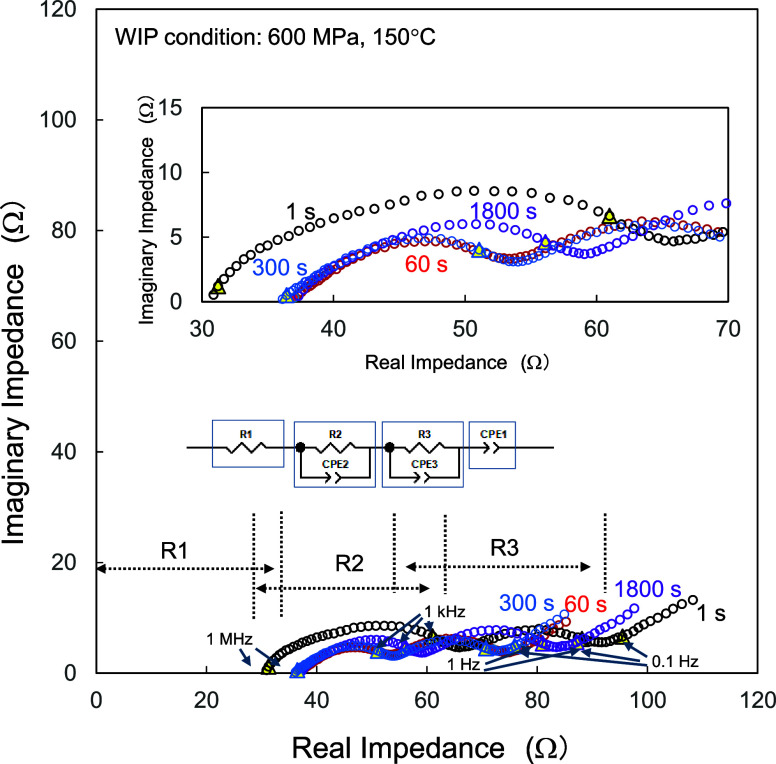
EIS results (Nyquist plots) of the corresponding test cells. The data were taken at a voltage of 3.3 V during charging. The inset shows the equivalent circuit used for the analysis. The Nyquist plots extracted from the high- and intermediate-frequency regions are also displayed.

The data were analyzed using the equivalent circuit shown in the inset of Figure 6, including the bulk resistance (R1) calculated from the offset from the origin. Figure 7 summarizes the values of R1, R2, and R3. The values of R1 were in the range 30–40 Ω. R1 was likely induced by the structure of the test cell. More precisely, the offset from the origin on the horizontal axis was due to the existence of bulk (Ohmic) resistance from the pellet-shaped SEs and at the interfaces between the composite cathodes and pellet-shaped SEs.

**Figure 7:**
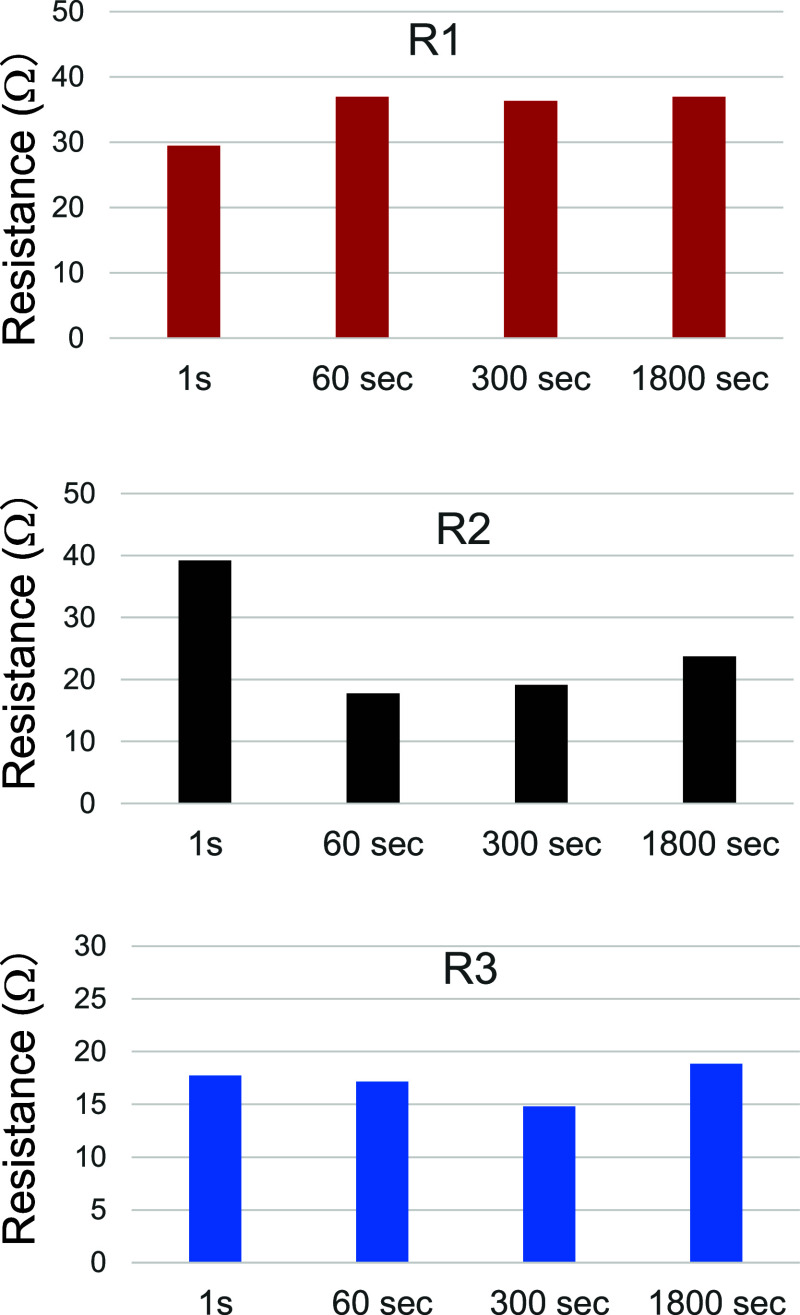
Summary of R1, R2, and R3 values. The data were analyzed using the equivalent circuit shown in the inset of Figure 6.

The value of R2 for TCW1 is much higher than that for the other composite cathodes. The values of R2 significantly decreased with the process duration and then increased slightly. In contrast, R3 had less impact on the electrochemical performance of the cathodes because the value of R3 remained almost constant, regardless of the process duration. The fact that both the void ratio and R2 values showed the same trends as the process duration suggests that R2—thus, the charge transfer at the interface—is the dominant factor in determining the performance of the ASSBs. The existence of voids at the interface causes a lack of secure interfacial properties or an adequate contact area, resulting in insufficient Li-ion conduction.

As mentioned above, R3 has less impact on the electrochemical performance of the cathodes but should be correlated with the void ratio, as shown in Figure 5, where the lowest value was obtained for TCW300. Figure 8 shows the Bode plots for TCW60, TCW300, and TCW1800. The Bode plots were obtained from the results of EIS before charging (∼3.0 V), during charging in the range of 3.3–3.5 V, and after charging (∼3.7 V). As per the inferences, R3 values depended on the measurement voltages, whereas R2 values remained unchanged. R3 increased monotonically with a decrease in the applied voltage. These results imply that the generation of an overpotential at the SE reduces the effective voltage applied to the AM. It can be reasonably assumed that the existence of voids limits the total Li-ion conduction in the SEs. The dips observed in the discharge curves above 100 mAh g^–1^ could be due to an increase in R3.

**Figure 8:**
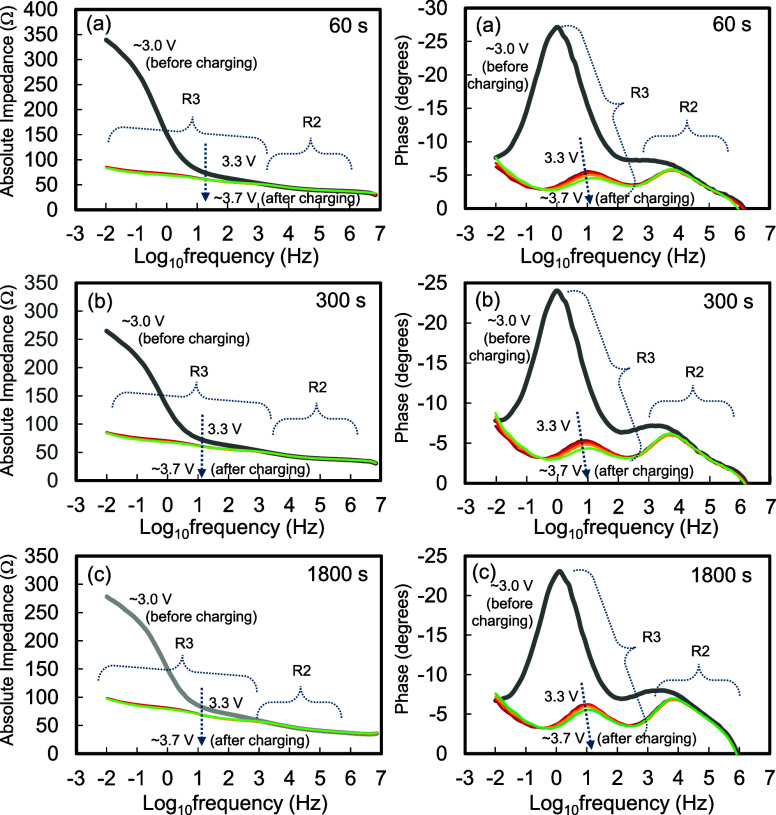
Bode plots of (a) CCW60, (b) CCW300, and (c) CW1800. The Bode plots have been calculated in the voltage range of around 3.0–3.7 V.

In our previous study, in the case of short-period pressing, such as 1 s WIP, the cathode instantly densified to some extent but remained suspended owing to contact with AM particles.^27^ Consequently, many microvoids remained in the SEs because of the release of the pressure surrounding the AM particles in contact. The suspension of densification commonly occurs in other short-period pressing methods, such as conventional roll pressing. With longer pressing schemes such as 60 s WIP, the voids in the SE gradually diminished with the enhanced rearrangement of the AMs and SEs. Therefore, the process duration, that is, the holding time under high isostatic pressure, is crucial for obtaining suitable interface properties between AMs and SEs.

However, a holding time longer than the optimum conditions causes microstructural degradation, since the rearrangement of the AM particles should be excessively enhanced during longer WIP treatments. This results in the aggregation of the AM particles and eventually causes the separation of the aggregated AMs and SEs. Figure 4d,h confirms the connection of the AM particles forming large, aggregated particles, and the number of the AM particles decreases after 1800 s of WIP. As the process continues, it is obvious that the enlargement of the AM particles reduces the contact area between the AMs and SEs. Therefore, the increase in the R2 values observed in the longer WIP treatment is due to the reduction of the contact area, rather than the degradation of the interfacial properties.

Furthermore, with the rearrangement of the AMs, a relatively large network structure forms in the cathodes. Once the network structure appears, by analogy with the case for the initial contact by pressing mentioned above, the applied pressure inside the network should be gradually released. At this stage, the SE particles do not follow the rearrangement of the AMs anymore, and the voids regenerate in the coarsened SEs. Relatively large voids can be seen in the upper middle and right corner of the SE region of Figure 4h. The change in the composite structures could affect the electrochemical responses in the higher-frequency region. However, the variation of the values of R1 estimated from the equivalent circuit in Figure 6 was small, and the contribution to the electrochemical response was negligible in the present study.

We also observed additional degradation modes that were enhanced in the low-temperature regime. Figure 9a shows the discharge performance of the test cells as a function of the process duration. All the composite electrodes were pressurized at 600 MPa and 120 °C via WIP. The specific capacities of the test cells decreased as the process duration increased. In accordance with the reduction in capacity, an additional shoulder was observed in the higher-frequency region in the Bode plots, as shown in Figure 9b. The plots were reproducible with the addition of a pure capacitance component. This result suggests that the degradation could be due to microgaps at the interface and/or cracks in the SEs. Cross-sectional SEM images revealed that voids remained near the interface region of the 120 °C samples (indicated by arrows in Figure 10a), which were nearly absent in the 150 °C WIP samples (Figure 10b). It is known that the argyrodite-type SEs gradually soften over 100 °C.^34^ In Figure 10b, most of the SEs coarsened to form one grain with good interfaces with the AM particles, which is hardly observed in Figure 10a. It is worth noting that the coalesced structure was hardly obtained with short-period pressing. Figure 10c shows the cross-sectional SEM image for the cathodes with the one s WIP treatment at 150 °C. It is obvious that even in the treatment at 150 °C, the AM particles were isolated and the contacts between the AMs and SEs were apparently not observed, causing the lower discharge performance evidenced in Figure 3.

**Figure 9:**
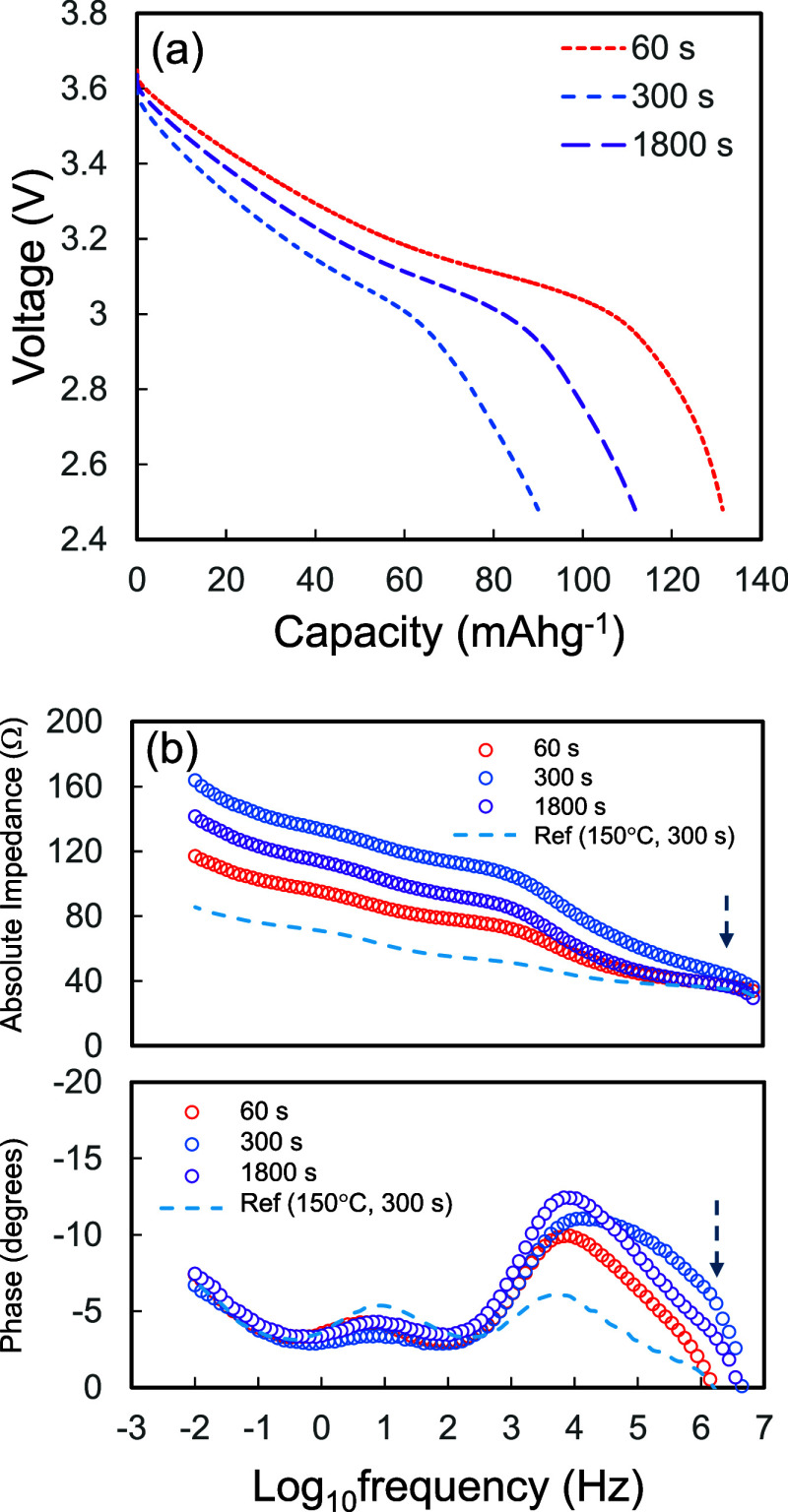
(a) Charge performance of the test cells as a function of the process duration. All the composite electrodes were pressurized at 600 MPa and 120 °C using WIP. (b) Bode plots of corresponding test cells. The data were obtained at a voltage of 3.3 V during charging. The data obtained from CCW300 are also plotted for comparison.

**Figure 10:**
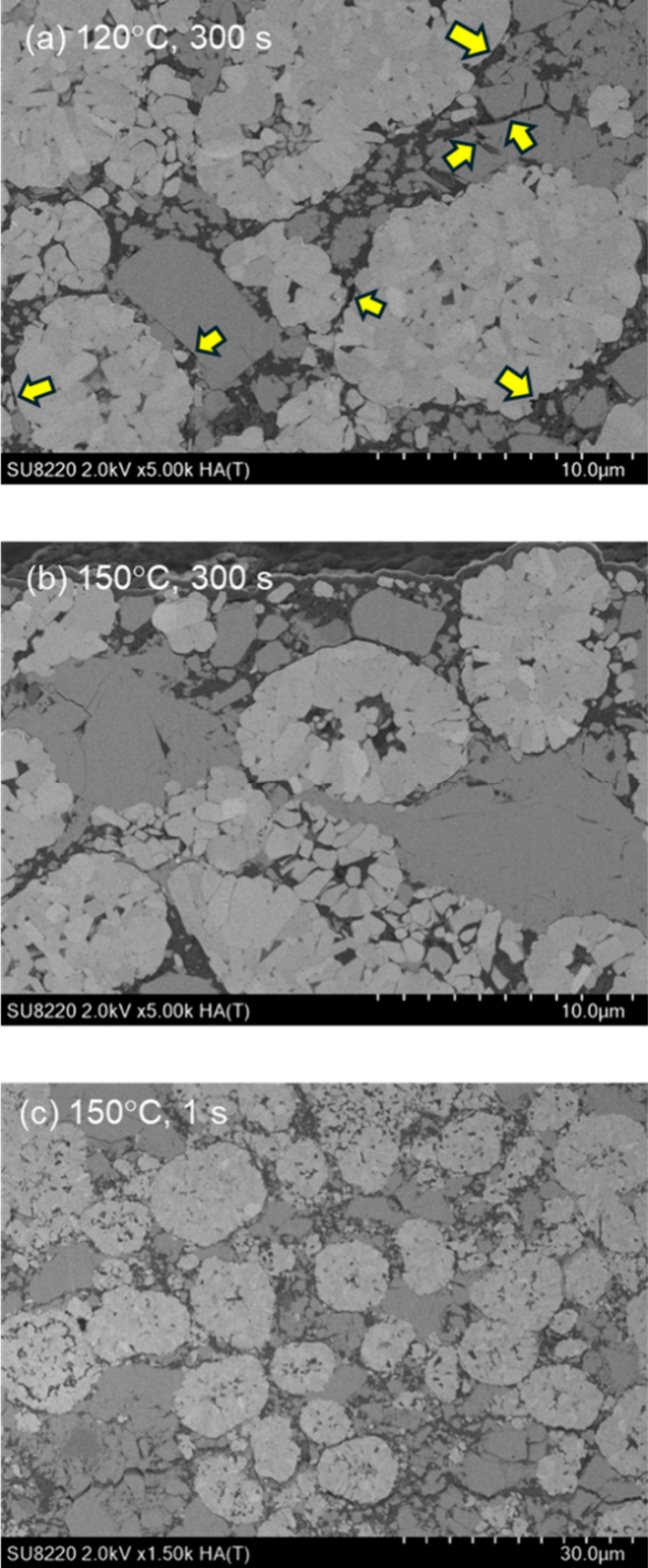
Cross-sectional SEM images of cathodes treated with WIP at (a) 120 °C for 300 s, (b) 150 °C 300 s, and (c) 150 °C for 1 s. Note that the magnification of (c) was lower than the others to see the distribution of the AM particles.

There remains a discrepancy in the trends between the EIS analysis and the charge–discharge performances. The charge transfer resistance of the 60 s WIP test device showed the lowest value, while the discharge capacity was slightly smaller than that for the 1800 s WIP case, having a higher R2 value. The void ratio for the 1800 s WIP case also showed higher than that for 60 s. From the arguments mentioned above, the increase in the R2 values should be attributed to the change in contact area. The interfacial properties improved and remained sufficient by the WIP for 60 s or more. In the case of the 1800 s WIP, the relatively large voids were located mainly in the SEs, not at the interfaces. Both factors could contribute to providing a higher discharge capacity than the other WIP cases. In addition, the total fraction of the area where the contacts at the interfaces between the AMs and SEs were not secured could decrease with the WIP duration. The SEM images shown in Figure 10b,c support the above argument. Further investigation is required to clarify these points. The present study also implies that the resultant structure after the WIP treatment is influenced by the original (i.e., as-fabricated) microstructure of the cathode. Without such rearrangements, the electrochemical performance would not be reproducible. Hence, a detailed investigation of the temperature dependence of the mechanical properties of each element is required.

## Conclusions

We performed a parametric study on the densification of composite cathodes treated with WIP to investigate the effect of the process duration on the electrochemical performance of the ASSBs. The EIS and charge–discharge performances were examined to determine the interfacial properties of the composite cathodes. The structures of the composite electrodes before and after WIP were examined via X-ray CT using synchrotron radiation to correlate the microstructure with the electrochemical performance of the composite cathodes.

It was evident that the specific capacity of the test cells increased with the process duration. Furthermore, the microstructure of the composite cathodes varied depending on the process duration. The void ratio for the 1 s WIP cathode was almost double that of the others, indicating that a large number of small voids remained in the SE region. Accordingly, the test cells with the 1 s WIP cathode showed two-thirds of the specific capacities of the others. We concluded that the resistance related to charge transfer is the dominant factor determining the performance of the ASSBs. In contrast, the Li intercalation/deintercalation process has less impact on the electrochemical performance of the cathodes but is correlated with the void ratio. In conclusion, the process duration, that is, the holding time under high isostatic pressure conditions, is crucial for obtaining suitable interface properties between the AM and SE. Densification using WIP is expected to be advantageous not only for improving the electrochemical performance of ASSBs but also for increasing the reproducibility of ASSB production.

It is notable that extensive research using different types of SEs having other elements and/or structures would be beneficial for obtaining overall comprehension that inherently exists in the characteristics of the interfaces. Application of analytical tools having atomic resolution is also effective to discuss the nature of the interfaces that govern the performance of the ASSBs.
